# Oral gel loaded with penciclovir–lavender oil nanoemulsion to enhance bioavailability and alleviate pain associated with herpes labialis

**DOI:** 10.1080/10717544.2021.1931561

**Published:** 2021-06-01

**Authors:** Khaled M. Hosny, Amal M. Sindi, Hala M. Alkhalidi, Mallesh Kurakula, Nabil K. Alruwaili, Nabil A. Alhakamy, Walaa A. Abualsunun, Rana B. Bakhaidar, Rahaf H. Bahmdan, Waleed Y. Rizg, Sarah A. Ali, Wesam H. Abdulaal, Majed S. Nassar, Mohammed S. Alsuabeyl, Adel F. Alghaith, Sultan Alshehri

**Affiliations:** a Department of Pharmaceutics, Faculty of Pharmacy, King Abdulaziz University, Jeddah, Saudi Arabia; b Center of Excellence for Drug Research and Pharmaceutical Industries, King Abdulaziz University, Jeddah, Saudi Arabia; c Oral Diagnostic Sciences Department, Faculty of Dentistry, King Abdulaziz University, Jeddah, Saudi Arabia; d Department of Clinical Pharmacy, Faculty of Pharmacy, King Abdulaziz University, Jeddah, Saudi Arabia; e Department of Biomedical Engineering, The Herff College of Engineering, Memphis, TN, USA; f Department of Pharmaceutics, Faculty of Pharmacy, Jouf University, Sakaka, Saudi Arabia; g Department of Biochemistry, Faculty of Science, Cancer and Mutagenesis Unit, King Fahd Center for Medical Research, King Abdulaziz University, Jeddah, Saudi Arabia; h Life Science and Environment Research Institute, King Abdulaziz City for Science and Technology (KACST), Riyadh, Saudi Arabia; i Department of Pharmaceutics, College of Pharmacy, King Saud University, Riyadh, Saudi Arabia; j Department of Pharmaceutical Sciences, College of Pharmacy, Almaarefa University, Riyadh, Saudi Arabia

**Keywords:** Self nanoemulsion, herpes labialis, lavender oil, oral gel, penciclovir, pseudoplastic

## Abstract

Herpes labialis, caused by herpes simplex virus type 1, is usually characterized by painful skin or mucosal lesions. Penciclovir (PV) tablets are found to be effective against herpes labialis but suffer from poor oral bioavailability. This study aimed to combine the benefits of PV and lavender oil (LO), which exhibits anesthetic activity, in the form of a self-nanoemulsifying drug delivery system (SNEDDS) for the treatment of herpes labialis. Toward this purpose, LO (oil), Labrasol:Labrafil M1944 CS in the ratio of 6:4 (surfactant mixture), and Lauroglycol-FCC (co-surfactant, selected based on the solubility of PV) were evaluated as the independent factors using a distance quadratic mixture design. The formulation was optimized for the minimum globule size and maximum stability index and was determined to contain 14% LO, 40.5% Labrasol:Labrafil 1944 (6:4), and 45.5% Lauroglycol-FCC. The optimized PV-LO-SNEDDS was embedded in chitosan hydrogel and the resulting formulations coded by (O3) were prepared and evaluated. The rheological studies demonstrated a combined pseudoplastic and thixotropic behavior with the highest flux of PV permeation across sheep buccal mucosa. Compared to a marketed 1% PV cream, the O3 formulation exhibited a significantly higher and sustained PV release, nearly twice the PV permeability, and a relative bioavailability of 180%. Overall, results confirm that the O3 formulation can provide an efficient delivery system for PV to reach oral mucosa and subsequent prolonged PV release. Thus, the PV-LO-SNEDDS embedded oral gel is promising and can be further evaluated in clinical settings to establish its therapeutic use in herpes labialis.

## 1. Introduction

Oral health is considered a major constituent of overall health and involves much more than having healthy teeth. While the mouth harbors numerous harmless bacteria under control, improper oral care can consequently lead to uncontrolled growth of bacteria and subsequent tissue damage, resulting in tooth and gum impairment. In addition, some diseases, such as diabetes, HIV/AIDS, and osteoporosis, can adversely affect oral health, which could further lead to other issues for instance endocarditis, cardiovascular diseases, pregnancy and birth complications, and pneumonia (Li et al., 2000). Infections in the oral cavity can be caused by bacteria, fungi, or viruses (Bandara & Samaranayake, 2019). Among these, viral infections are less common but are manifested as ulcers or blisters that can be detrimental and hence lead to poor quality of life.

Herpes labialis, or cold sore, is a viral infection caused by herpes simplex virus type 1 (HSV-1) and is usually characterized by painful skin or mucosal lesions (Santosh & Muddana, 2020). Several therapeutic strategies have been proposed against herpes labialis along with both short-term and long-term preventive treatments. Amongst the commonly used, both acyclovir and penciclovir (PV) creams are found to be effective against herpes labialis (Opstelten et al., 2008). Topical applications of zinc oxide or zinc sulfate, anesthetic, and antiviral creams can achieve good results upon prompt use, among which antiviral creams are considered the most effective therapeutic strategy.

PV has been proven to be effective against both HSV-1 and HSV-2 in recurrent herpes labialis (Spruance et al., 1997). Compared to acyclovir and valacyclovir creams, PV cream has been reported to significantly reduce the time of lesion healing and decrease lesion area and pain. One major advantage of PV is that its active form shows a very long half-life in HSV-infected cells (Schmid-Wendtner & Korting, 2004). Nevertheless, since it has a poor oral bioavailability of 5–10%, topical formulations with liposomes, microemulsion, and microemulsion gels have been proposed (Yang & Wang, 2005; Zhu et al., 2008, 2009). Further, these advanced delivery systems can also reduce the adverse effects of PV (Zhu et al., 2009).

The formulation components of micro- and nanoemulsions contribute to the enhancement of drug bioavailability from these advanced nanostructured delivery systems (Hosny et al., 2021a, b). Mainly, the oil phase for these formulations is selected based on its power to solubilize the drug and, less frequently, on the therapeutic purpose of the system. In this context, essential oils are ideal for providing the oil phase and additional antiviral activity, specifically against HSV-1 (Hosny et al., 2020; Alghaith et al., 2021). It is pertinent to mention that lavender oil (LO) from *Lavandula* plants (Lavender) shows significant antiviral activity (Alghaith et al., 2021), and LO creams have demonstrated successful treatment against herpes labialis lesions (Altaei & Ahmed, 2012). Moreover, the anesthetic activity of LO can be of immense use in alleviating pain of lesions in herpes labialis (Ghelardini et al., 1999).

Noteworthy, the chemical constituents of lavender vary depending on its variety (Białoń et al., 2019) and primarily include linalool, linalyl acetate, lavandulol acetate, and β-caryophyllene oxide (Dong et al., 2020). Compounds, such as α-terpineol, 4-terpinenol, and linalool, have shown specific antiviral activity (Król et al., 2013), while the local anesthetic activity of linalool and linalyl acetate from LO has been established (Koulivand et al., 2013). Additionally, 4-terpinenol, thymol, and carvacrol are known to exert anti-inflammatory actions, providing additional benefits against herpes labialis (Król et al., 2013).

Self-nanoemulsifying drug delivery systems (SNEDDS) are widely used to enhance the solubility and bioavailability of drugs with poor aqueous solubility. On dilution with a physiological medium, SNEDDS produce nano-sized drug-loaded oil droplets that are primarily responsible for the improved absorption and bioavailability (Cherniakov et al., 2015), which is least affected by the presence or absence of food (Charman et al., 1993). Furthermore, SNEDDS offer high physical and chemical stabilities and can be converted into other dosage forms, such as tablets and capsules (Shahba et al., 2012). Therefore, the use of LO as an oil phase to formulate PV-loaded SNEDDS would be a promising strategy to explore the combined effects of these components.

Considering that micro- and nanoemulsion-based systems for oral application suffer from poor residence in the oral cavity, adequate retention of the PV-loaded system is essential for effective therapy of herpes labialis. Fortunately, oral gels can satisfactorily solve this issue by providing sustained or controlled release of therapeutic agents for an enhanced effect (Miyazaki et al., 2009; Aslani et al., 2016). Since prolonged exposure of herpes labialis lesions to PV can improve therapeutic efficacy, incorporating SNEDDS in a gel base would greatly offer an additional advantage.

The formulation of SNEDDS and oral gels is multifactorial process, involving the drug, excipients, and methods. However, the process of selecting the optimal formula using conventional experiments can be highly complex and time-consuming. Comparatively, the design of experiments approach which involves simultaneous and systematic evaluation of the formulation or process variables with the minimum number of experiment trials, offers distinct advantages and reaching the optimum formula rapidly (N Politis et al., 2017).

Therefore, this study aimed to formulate PV-loaded LO (PV-LO) in the form of SNEDDS (PV-LO-SNEDDS) by employing a distance quadratic mixture design and its subsequent incorporation in an oral gel. It was anticipated that the optimized PV-LO-SNEDDS-loaded oral gel (O3) would enhance the bioavailability of PV, whereas the use of LO as an oil phase would contribute to the anesthetic activity and may also alleviate pain associated with herpes labialis. Most importantly, the incorporation of the optimized PV-LO-SNEDDS within the oral gel base would ensure intimate contact with oral mucosa, and support the prolonged release of PV to relieve herpes labials.

## 2. Materials and methods

### 2.1. Materials

PV was obtained as a gift sample from Qingdao Sigma Chemical Co., Ltd. Shandong, China. Basil oil, thyme oil, peppermint oil, rosemary oil, LO, citronella oil, verbena oil, and camphor oil were procured from Jiangxi Origin Aromatics Co., Ltd. Jiangxi, China. Tween80, Span80, Steareth-2, Steareth-21, Labrasol, Labrafil 1944, Brij 30, Lauroglycol-FCC, Lutrol-E400, propylene glycol, and Transcutol were obtained as gift samples from Gattefosse (Saint-Priest, France).

### 2.2. Estimation of PV solubility in various SNEDDS components

In this study, all previously-mentioned essential oils were studied for the solubility of PV. Meanwhile, Tween80:Span80 in the ratio 5.33:4.67, Steareth-2: Steareth-21 in the ratio 4.9:5.1, Labrasol:Labrafil 1944 in the ratio 6:4, and Brij 30 were evaluated for the solubility of PV to use as the surfactants in the proposed SNEDDS. As the reported HLB for LO is 10 so the different pairs of surfactant in each surfactant mixture were mixed in ratios which allow the HLB for the mixture equal 10. In addition, Lauroglycol-FCC, Lutrol-E400, propylene glycol, and Transcutol were studied as co-surfactants. The solubility of PV was assessed by shaking an excess of PV with 2 mL of the tested samples at 25 ± 0.5 °C for 48 h. The samples were then centrifuged (1200 rpm, 15 min), diluted with methanol, and quantified by UV–Vis spectrophotometry at 260 nm.

### 2.3. Pseudoternary phase diagram in various solvent systems

A pseudoternary phase diagram for locating the nanoemulsion region with LO (oil phase), Labrasol:Labrafil 1944 in the ratio 6:4 (surfactant mixture), and Lauroglycol-FCC (co-surfactant) was prepared. The total composition of the three components was maintained at 100% with 100 mg PV. The definite compositions were prepared for the identification of the nanoemulsion region in the diagram.

### 2.4. Preparation and optimization of PV-LO-loaded SNEDDS

In order to optimize compositions of the selected oil, surfactant, and co-surfactant phases, a distance quadratic mixture design was used. Three independent variables were studied for this purpose: Factor A was LO in the range of 11–25%; Factor B was the surfactant mixture (Labrasol:Labrafil 1944) in the range of 39–53%; and Factor C was Lauroglycol-FCC co-surfactant in the range of 36–50%. The globule size and stability index were taken as the responses for the evaluation and optimization of the PV-LO loaded SNEDDS formulation. A total of 15 runs were executed randomly and contained 100 mg PV in each formulation (Table 1). About 1 g of each mixture was prepared by simply mixing the three components (oil, surfactant, and co-surfactant).

**Table 1.** The independent factors and responses for the design of experiments.

Run	Coded values			Actual values		
	Factor A	Factor B	Factor C	Factor A	Factor B	Factor C
	Lavender oil	Surfactant mixture	Lauroglycol-FCC	Lavender oil (%)	Surfactant mixture (%)	Lauroglycol-FCC (%)
1	0.157322	0.436232	0.406446	15.7	43.6	40.7
2	0.11	0.42556	0.46444	11.0	42.6	46.4
3	0.25	0.39	0.36	25.0	39.0	36.0
4	0.179863	0.460137	0.36	18.0	46.0	36.0
5	0.145034	0.39	0.464966	14.5	39.0	46.5
6	0.179478	0.39	0.430522	17.9	39.0	43.1
7	0.11	0.460572	0.429428	11.0	46.1	42.9
8	0.11	0.39	0.5	11.0	39.0	50.0
9	0.11	0.53	0.36	11.0	53.0	36.0
10	0.133466	0.483083	0.38345	13.3	48.3	38.4
11	0.11	0.39	0.5	11.0	39.0	50.0
12	0.25	0.39	0.36	25.0	39.0	36.0
13	0.11	0.53	0.36	11.0	53.0	36.0
14	0.204559	0.412952	0.382489	20.5	41.3	38.2
15	0.179863	0.460137	0.36	18.0	46.0	36.0

#### 2.4.1. Preparation of PV-LO-loaded SNEDDS

The PV-LO-SNEDDS formulations were prepared by mixing and vortexing the specified proportions of the LO (oil), Labrasol:Labrafil 1944 in the ratio 6:4 (surfactant mixture), and Lauroglycol-FCC (co-surfactant) for 5 min. Finally, the mixture was allowed to achieve equilibrium for 12 h in a shaker water bath set at 100 rpm and 37 °C.

#### 2.4.2. Evaluation of the PV-LO-loaded SNEDDS

##### 2.4.2.1. Determination of globule size of the PV-LO SNEDDS

The aqueous dispersions of the PV-LO SNEDDS obtained after each trial were subjected to 10 times dilution with distilled water, then the diluted samples were evaluated for globule size (Zetatrac, Microtrac, Montgomeryville, PA).

##### 2.4.2.2. Determination of stability index

The stability index was estimated by exposing the PV-LO-SNEDDS formulations to consecutive freeze-thaw cycles thrice at a freezing temperature of −25 °C for 12 h and thaw temperature of 25 °C for 12 h. The stability index was determined using the initial and final globule sizes using Equation (1) (Sindi et al., 2021):

(1) $$\text{Stability index of SNEDDS }=\frac{\text{Original globule size }–\text{ Change in globule size}}{\text{Original globule size}}\times 100$$

#### 2.4.3. Optimization of PV-LO-loaded SNEDDS

The optimization of the formulation was performed by selecting the optimal percentages of LO (Factor A), surfactant mixture (Factor B), and Lauroglycol-FCC (Factor C). The minimum value for globule size and maximum value for stability index were analyzed as the constraints on response variables.

### 2.5. Development of the chitosan oral gel loaded with PV-LO-SNEDDs

The chitosan-based oral gel was prepared by loading of PV-LO-SNEDDs into a chitosan hydrogel. The hydrogel was first prepared by dispersing 200 mg chitosan in 10 mL of 1.5% dilute aqueous acetic acid, then stirring at 500 rpm and 25 ± 1 °C. Afterward, the required quantity of optimized PV-LO-SNEDDS was dispersed into the chitosan hydrogel with a final PV concentration of 1%. Other hydrogel samples were also prepared in a similar manner (Table 2) and stored overnight under refrigeration until further use (Sindi et al., 2021).

**Table 2.** Compositions of the different chitosan oral gel samples/formulations.

Formulation	Composition
O1	Chitosan hydrogel (plain)
O2	Chitosan hydrogel loaded with PV powder
O3	Chitosan hydrogel loaded with optimized PV-LO-SNEDDs

### 2.6. Rheological characterization of the chitosan oral gel loaded with PV-LO-SNEDDs

The rheology of chitosan oral gel loaded with PV-LO-SNEDDs (O3) was compared with plain chitosan hydrogel (O1). Samples (1 g each) were evaluated at 25 ± 1 °C (Brookfield viscometer, spindle 52) at shear rates of 2, 10, 20, 30, 40, 50, and 60 s^−1^ to obtain the flow curves. The viscosities at a maximum (η_max_) and minimum (η_min_) rate of shear were determined according to a previous study (Sindi et al., 2021), and Farrow’s constant (n) was calculated using Equation (2):

(2) Log G =n Log F–Log ɳ

where G is the shear rate; *ɳ* is viscosity; *F* is the shear stress; and *n* is Farrow’s constant.

### 2.7. *In vitro* release of the PV-LO-SNEDDS-loaded chitosan oral hydrogel

The release of PV from the O2, O3, marketed PV cream (1%), and 1% PV aqueous suspension was studied using a modified Type I USP apparatus in accordance with a reported procedure (Sindi et al., 2021). In this work, the previously activated membrane (100-µm pore size) was tied to the lower part of a cylindrical tube (length of 10 cm and diameter of 2.7 cm), instead of a basket. Accurately weighed samples containing 10 mg PV were placed inside the tubes, then the apparatus was set at 50 rpm and 37 ± 0.5 °C with phosphate-buffered saline (pH 6.8, 250 mL) as the medium. The dissolution samples were withdrawn at predetermined time intervals; 0.25, 0.5, 1.0, 1.5, 2.0, 2.5, and 3.0 h with replenishment of lost medium volume after each sampling with the fresh medium. The filtered (0.45 µm) samples were analyzed using high-performance liquid chromatography. Methanol mixed with 0.1 M ammonium acetate buffer at pH 6.0 in a volume ratio of 1:10 was used as the mobile phase. The flow rate of the mobile phase through the 5-μm ODS2 column at 35 °C was 1 mL/min and detected at 260 nm.

### 2.8. *Ex vivo* mucosal permeation studies

A reported procedure was followed to study the *ex vivo* mucosal permeation (Sindi et al., 2021). Fresh sheep buccal mucosa of dimension 2 × 2 cm with a diffusion area of 1.75 cm^2^ was used as the membrane barrier in the Franz diffusion cell containing 8 mL of phosphate-buffered saline at pH 6.8 and 37 ± 1 °C as the receptor medium. The mucosal permeation of PV from O2, O3, marketed PV cream (1%), and 1% PV aqueous dispersion was determined. Samples were collected and the PV content was determined by HPLC. The filtered (0.45 µm) samples were analyzed using high-performance liquid chromatography. Methanol mixed with 0.1 M ammonium acetate buffer at pH 6.0 in a volume ratio of 1:10 was used as the mobile phase. The flow rate of the mobile phase through the 5-μm ODS2 column at 35 °C was 1 mL/min and detected at 260 nm. The cumulative PV permeation, steady-state flux (Jss), permeability coefficient (PC), enhancement factor (EF), relative permeation rate (RPR), and diffusion coefficient (D) were determined for the samples.

### 2.9. *In vivo* evaluation of the optimized PV-LO SNEDDs loaded chitosan oral hydrogel

The *in vivo* study was performed on three groups of male Wistar rats (200–250 g), which was approved by the local Institutional Review Board for Preclinical & Clinical Research (Approval No. 22-04-21). Group 1 orally ingested the O2 formulation at a PV dose of 10 mg/kg body weight. In Group 2, the animals were orally administered the optimized PV-LO SNEDDs-loaded chitosan oral hydrogel (O3) at a PV dose of 10 mg/kg. Group 3 animals were administered transdermal application of marketed PV 1% cream at a PV dose of 10 mg/kg. Finally, the pharmacokinetic parameters of PV including maximum plasma level (C_max_), time to reach maximum plasma level (t_max_), area under plasma concentration–time curve (AUC), and elimination rate constant (K), were determined (Kinetica version 4, Thermo Electron Corporation, Waltham, MA) and compared.

## 3. Results and discussion

### 3.1. Estimation of PV solubility in various SNEDDS components

Various essential oils, including basil, thyme, peppermint, rosemary, lavender, citronella, verbena, and camphor, were studied on the basis of their reported anesthetic activity, under the presumption that they exert a beneficial effect against pain associated with herpes labials. Such an advantage of essential oils has been proven in nanostructured carriers (Lai et al., 2007). The PV solubility in these oils was measured in an attempt to determine the most appropriate oil phase to be used. Table 3 indicates that all the essential oils exhibited good solubility of PV; this is in agreement with a report denoting that essential oils enhance the solubility of poorly water-soluble drugs (Hosny et al., 2019). Among the studied oils, PV was found to have a significantly higher solubility in LO with a value of 445 ± 17 mg/mL compared to the other oils (*p* value <.05). Therefore, LO was selected as the oil phase in the proposed PV-loaded SNEDDS. Given the fact that the required HLB for LO is 10, surfactant mixtures that provide an HLB value of 10 were studied. Amongst the surfactants studied, PV was found to have significantly higher solubility of 105 ± 9 mg/mL in Labrasol:Labrafil 1944 (6:4) (*p* value <.05), as shown in Table 3. Comparing all co-surfactants, Table 3 further indicates that Lauroglycol-FCC demonstrated a significantly higher solubility (79 ± 4 mg/mL; *p* value <.05). Therefore, LO, Labrasol:Labrafil 1944 (6:4), and Lauroglycol-FCC were selected as the oil phase, surfactant mixture, and co-surfactant, respectively, for the formulation of PV-loaded SNEDDS. In a similar study, a system containing Labrafil 1944, Labrasol, and Lauroglycol-FCC was reported to enhance the bioavailability of Coenzyme Q10 in a SNEDDS formulation (Balakrishnan et al., 2009). Therefore, the components of the proposed PV-LO-SNEDDS appeared promising for further screening and optimization.

**Table 3.** Solubility data of penciclovir (PV) in SNEDDS formulation components.

Component	Solubility of penciclovir (mg/mL)
Basil oil	210 ± 15
Thyme oil	320 ± 19
Peppermint oil	250 ± 11
Rosemary oil	95 ± 10
Lavender oil	445 ± 17
Citronella oil	170 ± 11
Verbena oil	200 ± 15
Camphor oil	154 ± 18
Tween80:Span80 (5.33:4.67)	67 ± 3
Steareth-2: Steareth-21 (4.9:5.1)	85 ± 8
Labrasol:Labrafil 1944 (6:4)	105 ± 9
Brij 30	52 ± 4
Lauroglycol-FCC	79 ± 4
Lutrol-E400	50 ± 5
Propylene glycol	65 ± 3
Transcutol	55 ± 7

### 3.2. Pseudoternary-phase diagram

**Figure 1.**
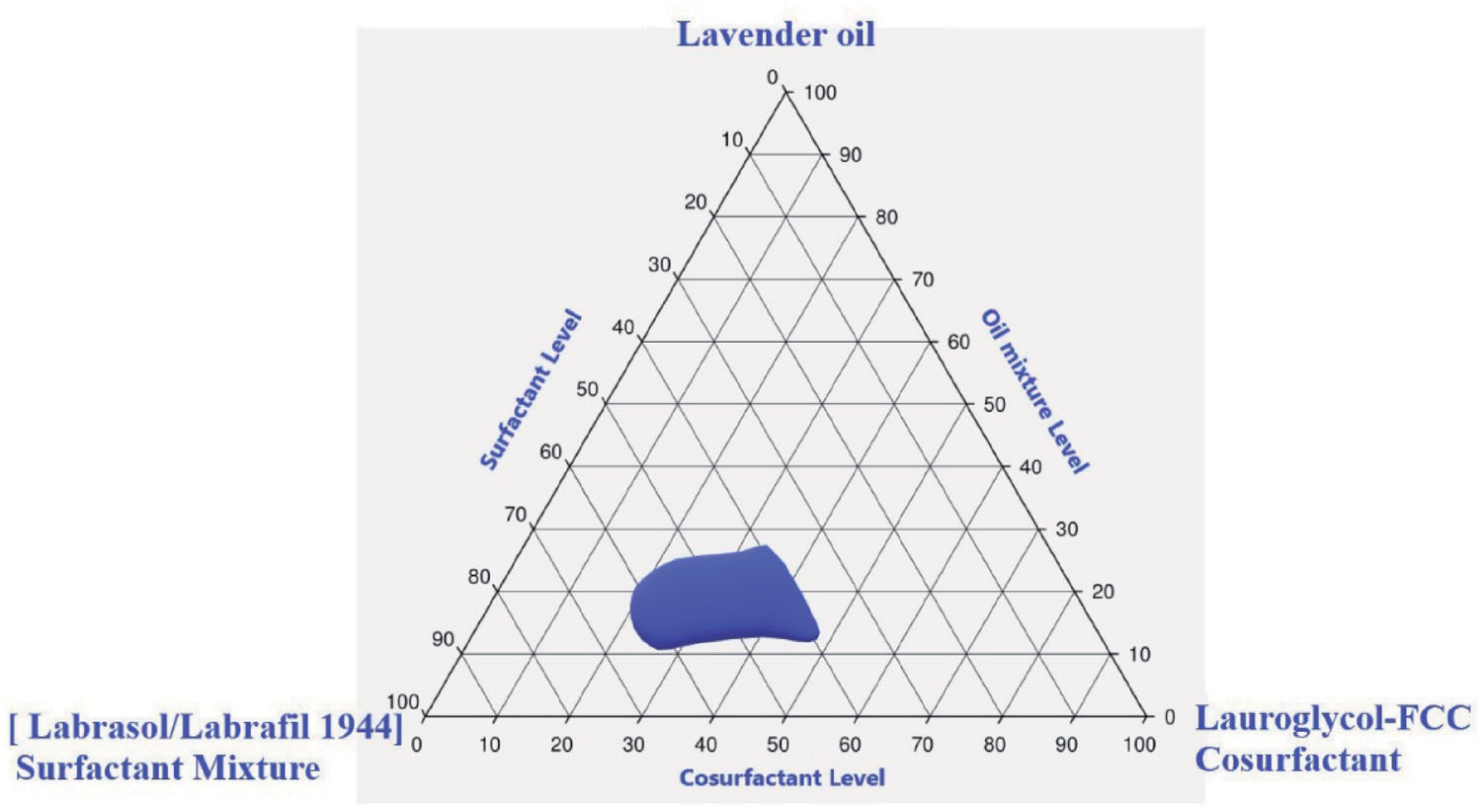
Pseudoternary-phase diagram in formulation components of penciclovir-loaded lavender oil-containing self-nanoemulsifying drug delivery systems (PV-LO-SNEDDS), using lavender oil (LO) as the oil phase, Labrasol:Labrafil 1944 in the ratio of 6:4 (HLB value = 10) as the surfactant mixture, and Lauroglycol-FCC as the co-surfactant.

Identification of the suitable levels for the formulation of PV-loaded SNEDDS was carried out by constructing a pseudoternary-phase diagram (Figure 1). The nanoemulsion region was observed when the LO concentration ranged between 11% and 27%, Labrasol:Labrafil 1944 (6:4) surfactant mixture level between 39% and 62%, and Lauroglycol-FCC level between 36% and 61%.

### 3.3. Preparation and optimization of PV-LO loaded SNEDDS

Table 4 provides the obtained globule size and stability index for each run suggested by the software.

**Table 4.** The independent factors and responses for the design of experiments.

Run	Factor A	Factor B	Factor C	Response 1	Response 2
	Lavender oil (%)	Surfactant mixture (%)	Lauroglycol-FCC (%)	Globule size (nm)	Stability index (%)
1	15.7	43.6	40.7	155	86
2	11.0	42.6	46.4	320	90
3	25.0	39.0	36.0	312	71
4	18.0	46.0	36.0	219	75
5	14.5	39.0	46.5	239	91
6	17.9	39.0	43.1	223	88
7	11.0	46.1	42.9	319	87
8	11.0	39.0	50.0	295	93
9	11.0	53.0	36.0	250	76
10	13.3	48.3	38.4	200	83
11	11.0	39.0	50.0	294	93
12	25.0	39.0	36.0	313	72
13	11.0	53.0	36.0	252	77
14	20.5	41.3	38.2	287	82
15	18.0	46.0	36.0	219	75

#### 3.3.1. Globule size

A special quadratic model was suggested for the globule size response with a model F value of 6409.04, and the lack of fit was not significant. Furthermore, adequate precision was acceptable with a value of 254.829, which is sufficiently large to use the design space. The analysis of variance (ANOVA) data for the globule size of PV-LO-SNEDDS is provided in Table 5.

**Table 5.** ANOVA data for globule size of PV-LO-SNEDDS prepared in various runs.

Source	Sum of squares	Degrees of freedom	Mean square	F-value	*p* Value	–
Model	35660.23	8	4457.53	6409.04	<.0001	Significant
Linear Mixture	4226.42	2	2113.21	3038.37	<.0001	–
AB	5242.72	1	5242.72	7537.99	<.0001	–
AC	6779.88	1	6779.88	9748.12	<.0001	–
BC	2334.13	1	2334.13	3356.02	<.0001	–
A²BC	3514.40	1	3514.40	5053.01	<.0001	–
AB²C	716.35	1	716.35	1029.96	<.0001	–
ABC²	2943.63	1	2943.63	4232.36	<.0001	–
Residual	4.17	6	0.6955	–	–	–
Lack of fit	1.17	2	0.5865	0.7820	.5168	Not significant
Pure error	3.00	4	0.7500	–	–	–
Cor total	35,664.40	14	–	–	–	–

**Figure 2.**
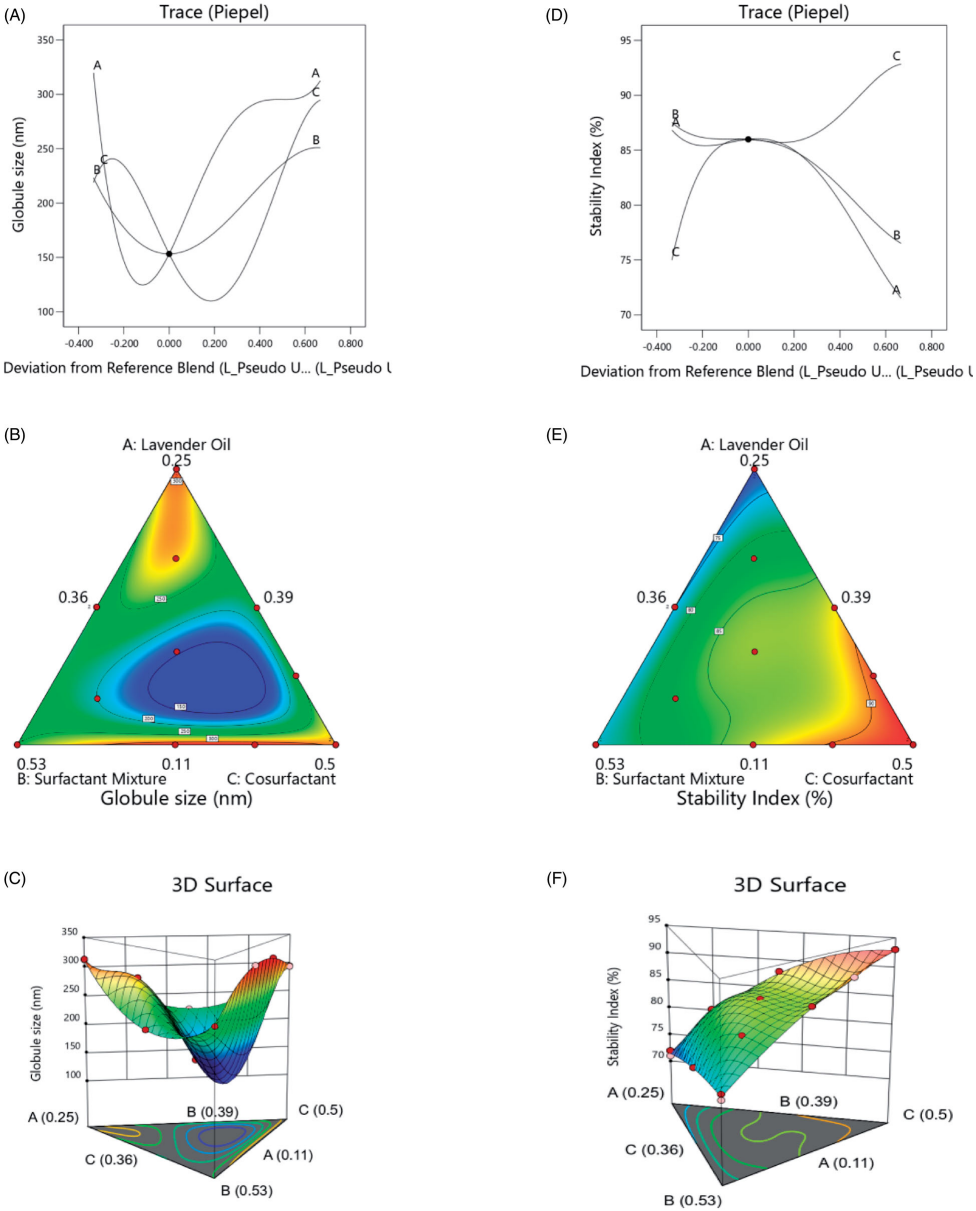
Various design plots for the globule size and stability index of PV-LO-SNEDDS: (a) trace plot for globule size, (b) simplex contour plot for globule size, (c) 3D plot for globule size, (d) trace plot for stability index, (e) simplex contour plot for stability index, and (f) 3D plot for stability index.

**Figure 3.**
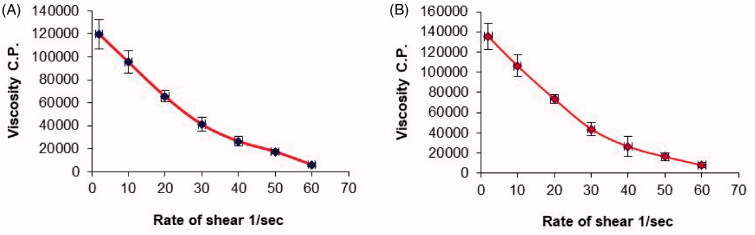
Plots of the shear rate (G) *versus* the viscosity (η) for: (a) plain chitosan oral hydrogel (O1) and (b) chitosan oral hydrogel loaded with the optimized PV-LO SNEDDs (O3). Values are expressed as mean ± SD (*n*= 3).

Globule size was calculated using Equation (3) based on the L-pseudo components. The trace plot was obtained to determine the effect of the individual design components on the response and, therefore, serves as the perturbation plot of the non-mixture design. The response trace plot in Figure 2(a) reveals that LO (%) (Factor A) has the greatest influence and surfactant mixture (%) (Factor B) has the least influence on the globule size of PV-LO-SNEDDS. The 2D simplex contour (Figure 2(b)) and 3D (Figure 2(c)) plots also confirm the significant effects of LO (%) (Factor A) and Lauroglycol-FCC (%) (Factor C) on globule size. The interaction effects displayed in these plots show that minimum values for globule size were observed at low values of Factor A, intermediate values of Factor B, and high values of Factor C. It has been established that higher oil content leads to larger globule size, and hence, the present observation regarding Factor A is acceptable (Sakeena et al., 2011). In addition, co-surfactants have been reported to reduce the interfacial tension of globules to values below that produced by a surfactant alone (Gurram et al., 2015). Thus, the observed effect of Lauroglycol-FCC as a co-surfactant in the PV-LO-SNEDDS is justified. However, the negligible effect of Labrasol:Labrafil 1944 (ratio of 6:4) as the surfactant mixture was rather unexpected. While high HLB surfactants generally decrease the globule size of o/w type nanoemulsions, surfactants with low HLB values have been proven to increase the globule size (Niamprem et al., 2014). Importantly, low HLB-value surfactants provide poor spreading and emulsification properties compared to high HLB-value surfactants (Eid et al., 2014). The very low Labrafil 1944 HLB of 3 (Jakab et al., 2018) and Labrafil 1944 oil-like properties (Balakrishnan et al., 2009), might be the reasons that contribute to the diminished effect of the surfactant mixture on globule size.

(3) $$\text{Globule}\text{size}=+312.49A+250.95B+294.69C-250.63\text{AB}-321.54\text{AC}+187.93\text{BC}+7228.{98A}^{2}\text{BC}-3204.{43\text{AB}}^{2}C-11323.{45\text{ABC}}^{2}$$

#### 3.3.2. Stability index

A special quadratic model was suggested for the globule size response with a model F value of 383.97, for which the lack of fit was not significant, and the adequate precision value was 52.6782. The analysis of variance (ANOVA) data for the stability index of PV-LO-SNEDDS are shown in Table 6.

**Table 6.** ANOVA data for stability index of PV-LO-SNEDDS prepared in various runs.

Source	Sum of squares	Degrees of freedom	Mean square	F-value	*p* Value	–
Model	837.96	8	104.75	383.97	<.0001	Significant
Linear Mixture	760.22	2	380.11	1393.37	<.0001	–
AB	1.25	1	1.25	4.60	.0758	–
AC	29.95	1	29.95	109.78	<.0001	–
BC	4.85	1	4.85	17.78	.0056	–
A²BC	6.25	1	6.25	22.93	.0030	–
AB²C	3.59	1	3.59	13.14	.0110	–
ABC²	3.23	1	3.23	11.83	.0138	–
Residual	1.64	6	0.2728	–	–	–
Lack of Fit	0.6368	2	0.3184	1.27	.3733	Not significant
Pure Error	1.0000	4	0.2500	–	–	–
Cor Total	839.60	14	–	–	–	–

The equation for the stability index derived in terms of the L-pseudo components is given in Equation (4). The response trace plot (Figure 2(d)) for the stability index further reveals that the effect of the factors follows the order: Lauroglycol-FCC (%) (Factor C) > LO (%) (Factor A) > surfactant mixture (%) (Factor B). The 2D simplex contour (Figure 2(e)) and 3D (Figure 2(f)) plots show that the stability index drastically increased when Factor C was increased. Considering that co-surfactants have been reported to increase the interfacial fluidity and disordering of a surfactant film (Isailović et al., 2017), a significant effect of Lauroglycol-FCC as the co-surfactant in PV-LO-SNEDDS on the stability index was anticipated. Moreover, the stability index was found to decrease when Factor A was increased, of note, the lowest stability index was obtained at the highest level of Factor A. Considering that higher oil content leads to larger globule size and subsequent reduction in droplet stability, such a decrease in stability index can be expected (Sakeena et al., 2011). The effect of surfactant mixture on stability index can be justified by the same explanations provided for its effect on the globule size, whereby the combination of both low and high HLB value surfactants exhibited an intermediate effect on the stability index.

(4) $$\text{Stability}\text{index}=+71.53A+76.52B+92.84C+3.88\text{AB}+21.37\text{AC}+8.57\text{BC}+304.{97A}^{2}\text{BC}+226.{71\text{AB}}^{2}C-374.{99\text{ABC}}^{2}$$

#### 3.3.3. Optimization of PV-LO-loaded SNEDDS

The optimum formula in Table 7 was found to contain 14% Factor A, 40.5% Factor B, 45.5% Factor C, and 100 mg PV. Then, the prepared PV-LO-SNEDDS using this optimized formula exhibited a globule size of 157 ± 9 nm and stability index of more than 88 ± 4%; values which are close to the predicted values.

**Table 7.** Optimum formula suggested for the PV-LO-SNEDDS formulation and the predicted and observed responses for the optimized formula.

Independent factors		
Factor	Variable	Level
A	Lavender oil (%)	0.1398
B	Surfactant mixture (%)	0.4048
C	Lauroglycol-FCC (%)	0.4554
Total =		1.0000
Responses		
	Predicted value	Observed value
Globule size (nm)	155	157 ± 9
Stability index (%)	87.8218	88 ± 4

### 3.4. Rheological evaluation of the PV-LO SNEDDs-loaded chitosan oral hydrogel

The rheological parameters of the chitosan hydrogel loaded with the optimized PV-LO-SNEDDS (O3) were determined in comparison to a plain chitosan hydrogel (O1) (Table 8). The rheological behavior of the formulations depends on both the material properties and process attributes (Ghica et al., 2016). The values of Farrow’s constant were more than 1 for both O1 and O3 samples, indicating a pseudoplastic behavior (El-Leithy et al., 2010). In this study, the chitosan hydrogel was prepared with 200 mg chitosan in 10 mL dilute aqueous acetic acid, corresponding to a concentration of 0.2 g/L. It has been reported that chitosan concentrations above 0.50 g/dL show a pseudoplastic or shear-thinning behavior (Kienzle-Sterzer et al., 1985). Thus, the pseudoplastic or shear-thinning properties of O1 and O3 gels can be justified. This was further confirmed from the plots of viscosity *versus* the rate of shear (Figure 3), whereby decreased viscosity of both samples was observed at higher shear rates, which is a characteristic of shear-thinning systems. Nonetheless, the minimum and maximum viscosities were higher for the O3 formulation compared to the O1 hydrogel. Previously, a published study has demonstrated the enhanced hydrogel viscosity after the inclusion of lipid-based nanostructures (Padamwar & Pokharkar, 2006), thereby, this may explain the higher viscosity of O3 with embedded SNEDDS compared to O1. Noteworthy, in addition to the advantage of the O3 formulation for sustaining PV release, also, the addition of lipid nanostructures will not prevent the gelation of chitosan but rather increases the rheological moduli by a significant proportion (Billard et al., 2015).

**Table 8.** Rheological parameters of plain chitosan hydrogel and chitosan hydrogel loaded with optimized PV-LO-SNEDDS.

Formulations	Viscosity* (maximum) (cP)	Viscosity* (minimum) (cP)	Farrow’s constant (N)	Flow behavior
Plain chitosan hydrogel (O1)	119,663 ± 9123	6123 ± 1075	3.718	Pseudoplastic
Chitosan hydrogel loaded with optimized PV-LO-SNEDDs (O3)	135,436 ± 12654	8112 ± 1548	4.117	Pseudoplastic

*Data are expressed as mean ± SD (*n* = 3).

**Figure 4.**
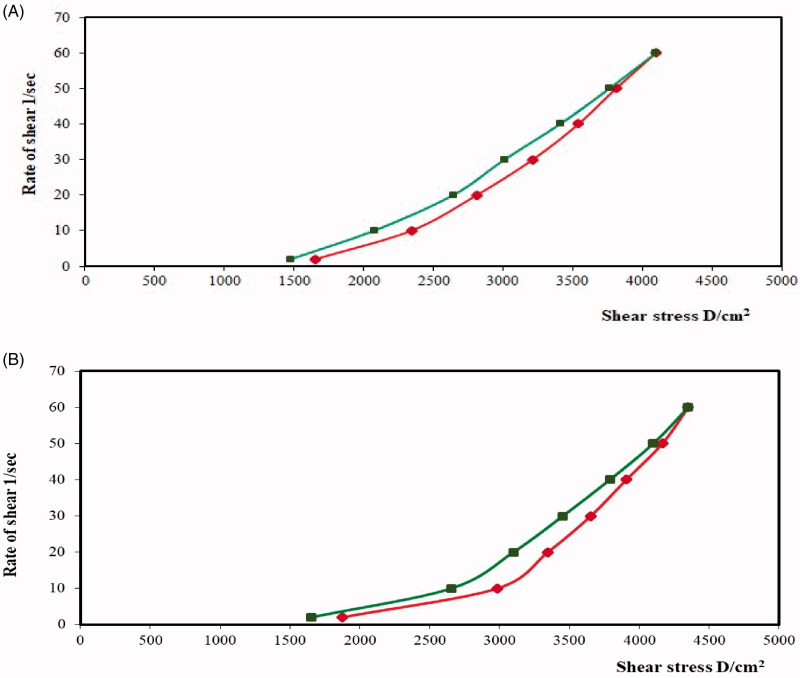
Rheograms of (**a**) plain chitosan oral hydrogel (O1) and (b) chitosan oral hydrogel loaded with the optimized PV-LO-SNEDDs (O3).

The thixotropy of the O1 and O3 formulations can be elucidated from the rheograms of the shear rate *versus* shear stress as shown in Figure 4. Specifically, the area of the formed loops represents the thixotropic behavior of the formulation (Ghica et al., 2016). Thus, the hysteresis loops of the O3 formulation exhibit a larger area than those of the plain chitosan hydrogel, thus it can be suggested that O3 has a slightly higher thixotropic behavior. This further implies that the O3 formulation with greater thixotropy can provide a better-sustained release of PV, easy application at the buccal mucosal surface, as well as an enhanced retention of the gel in the buccal mucosal surface (Lee et al., 2009).

**Figure 5.**
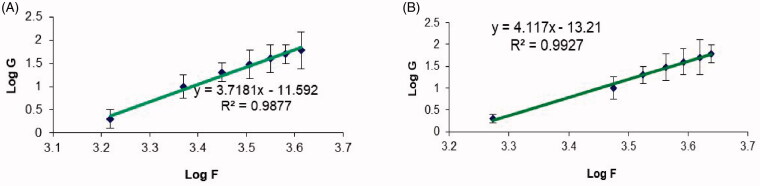
Plots of the logarithm of the shear rate (G) *versus* the logarithm of the shear stress (F) for (a) plain chitosan oral hydrogel (O1) and (b) chitosan oral hydrogel loaded with the optimized PV-LO-SNEDDs (O3). Values are expressed as mean ± SD (*n*= 3).

The plots for both shear rate and shear stress on logarithmic scales in Figure 5 provide a better delineation of the individual flow curves for further comparison (Jeong, 2019). Specifically, Farrow’s constant for the samples was determined from the slope of these plots. Thereafter, the rheological behaviors of the samples were evaluated based on the reciprocal of Farrow’s constant, or the slope of log shear stress *versus* log shear rate, whereby values less than one for the slope is indicative of a shear-thinning system (Wilkes, 1981). The results denote that O3 demonstrates a greater shear-thinning behavior compared to O1. Similarly, some evidence has demonstrated a combined pseudoplastic and thixotropic behavior for oral gel in a previous report (Hosny et al., 2019).

### 3.5. *In vitro* release of PV from PV-LO-SNEDDS loaded chitosan oral hydrogel (O3)

**Figure 6.**
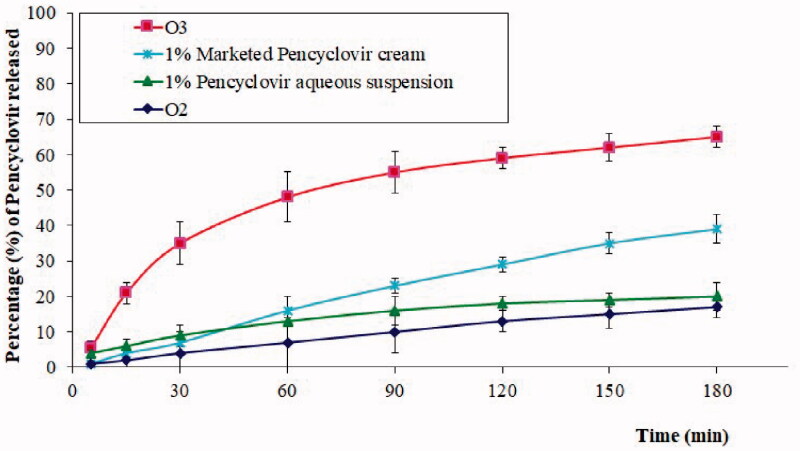
*In vitro* release profile of PV from chitosan hydrogel loaded with PV powder (O2), chitosan hydrogel loaded with optimized PV-LO SNEDDs (O3), marketed PV cream (1%), and 1% PV aqueous suspension. *Data are expressed as mean ± SD (*n* = 3).

A further comparative *in vitro* release study of the PV profile from O2, O3, commercial PV cream (1%), and 1% PV aqueous dispersion was carried out, and the results are shown in Figure 6. A significant increase, in addition to a sustained PV release was observed from the O3 compared to other samples. These observations can be described according to two aspects. First, the formulation of SNEDDS contributes to an enhanced drug dissolution and release of poorly soluble drugs (Kazi et al., 2020). Second, the polymer gel acts as a barrier for drug diffusion from the formulation into the medium, thus resulting in its release in a sustained manner (Xu et al., 2014). This effect is evident from the lower drug release from O2 compared to the 1% aqueous suspension of PV and the combined effect of enhanced and sustained release from the O3 formulation. Such a pattern is highly beneficial in certain conditions such as herpes labialis, wherein both enhanced concentration and a sustained release of PV are desired criteria. Meanwhile, other samples also showed a sustained release pattern, which could probably be explained due to the low dissolution and slow release of PV from these systems. The significantly higher PV release from O3 compared to the marketed PV cream revealed the importance of the O3 formulation in the management of herpes labialis.

### 3.6. Ex vivo mucosal permeation studies

*Ex vivo* permeability studies in sheep buccal mucosa confirmed that the O3 formulation significantly enhanced PV permeation (*p* value <.05) compared to all the other formulations (Table 9). The flux of permeation of PV followed the order: O3 > marketed PV (1%) cream > O2 > 1% PV aqueous suspension. Obviously, the PCs also showed the same order since the same dose of PV was loaded for all samples. The calculated RPR with respect to the marketed PV (1%) cream was also highest for O3 with a value of 2.069. Nevertheless, the O3 formulation increased PV permeation by 4.777 times compared to the 1% PV aqueous suspension, while the marketed PV (1%) cream could only enhance PV permeation by 2.308 times.

**Table 9.** Evaluation parameters of PV permeation across the buccal mucosa from different formulations.

Parameters of PV permeation*	Chitosan hydrogel loaded with optimized PV-LO-SNEDDs (O3)	Chitosan hydrogel loaded with PV powder (O2)	1% PV aqueous suspension	Marketed PV (1%) cream
Cumulative amount permeated (μg/cm^2^)	4911 ± 405	1402 ± 142	1028 ± 109	2373 ± 221
Steady state flux, Jss, (μg/cm^2^.min)	13.222	5.911	4.115	8.512
Permeability coefficient, PC, (cm/min)	11.5 × 10^−4^	4.7 × 10^−4^	3.6 × 10^−4^	8.6 × 10^−4^
Diffusion coefficient, D, (cm^2^/min)	39 × 10^−4^	15 × 10^−4^	11 × 10^−4^	26 × 10^−4^
Relative permeation rate (RPR)	2.069	0.59	0.433	–
Enhancement factor (EF)	4.777	1.363	–	2.308

*Data are expressed as mean ± SD (*n*= 3).

Interestingly, chitosan can improve the paracellular permeation of drugs across the epithelium of mucosa (Thanou et al., 2001a), which is facilitated by the reversible interaction of protonated chitosan with tight junction constituents that result in a widened paracellular path (Thanou et al., 2001b). This effect can be held responsible for the higher permeation of PV from O2 compared to the 1% aqueous suspension. Thus, the flux of permeation, permeation coefficient, diffusion coefficient, and RPR values were higher for O2 compared to the 1% aqueous suspension. In addition, essential oils like LO have demonstrated permeation-enhancing effects (de Matos et al., 2019), wherein linalool as a major constituent of LO can significantly influence permeation (Kamatou & Viljoen, 2008). Herein, only the O3 formulation contains LO; thus, the enhanced permeation of PV can be contributed to the presence of LO as the oil phase of SNEDDS. In addition, the SNEDDS formulation in O3 is also highly responsible for improving the permeation and spreadability of PV across buccal mucosa (Khan et al., 2018; Salem et al., 2019).

### 3.7. *In vivo* evaluation of the optimized PV-LO-SNEDDs loaded chitosan oral hydrogel

The pharmacokinetic data obtained from the *in vivo* studies are provided in Table 10, which demonstrates that the C_max_, AUC_0–t_, and AUC_0–inf_ were significantly higher (*p* value<.05) for O3 in comparison with the other formulations. Specifically, the increased C_max_ and AUC values of O3 can be contributed to the enhanced absorption of chitosan via the increased paracellular permeation of drugs across the mucosa epithelium (Thanou et al., 2001a,b). Due to the fact that the O2 formulation also contains chitosan, yet shows the least values for these pharmacokinetic parameters; this indicates that the increased C_max_ and AUC values of O3 could be attributed to the presence of SNEDDS. This result is in agreement with previous reports demonstrating the similar enhancement of C_max_ and AUC of poorly water-soluble drugs by the SNEDDS formulation (Yoo et al., 2013; Tripathi et al., 2016). Indeed, the enhanced and faster absorption results in the reduction of T_max_, which was observed for the O3 formulation with the lowest T_max_. When the drug is delivered as a sustained-release product, its elimination half-life increases and, subsequently, the k_el_ value is lowered (Kim et al., 2015). Therefore, the sustained delivery of PV from O3 was also confirmed by the lowest value of 0.534 ± 0.05 h^−1^ for this formulation. On the other hand, the relative bioavailability of the optimized O3 formula compared to the O2 oral gel and marketed PV 1% cream was found to be 245% and 180%, respectively. Importantly, these high relative bioavailability values confirm the efficacy of the O3 formulation in enhancing the bioavailability of PV.

**Table 10.** Comparative pharmacokinetic parameters of different PV formulations and marketed cream (mean ± SD, *n*= 6).

PK parameters	Chitosan hydrogel loaded with PV powder (O2)	Chitosan hydrogel loaded with optimized PV-LO-SNEDDs (O3)	Marketed PV 1% cream
C_max_ (ng/mL)	919 ± 112	2400 ± 290	1346 ± 205
T_max_ (min)	60 ± 30	30 ± 15	60 ± 15
AUC _0–t_ (ng/mL h)	3011.1 ± 222.1	7413.2 ± 698.3	4134.2 ± 543.4
AUC_0–inf_ (ng/mL h)	3922.1 ± 285.2	9564.5 ± 299.1	5611.2 ± 633.2
K_el_ (h^−1^)	0.655 ± 0.09	0.534 ± 0.05	0.605 ± 0.06

## 4. Conclusions

Based on the solubility studies, LO, Labrasol:Labrafil 1944 (6:4), and Lauroglycol-FCC were selected as the oil, surfactant mixture, and co-surfactant, respectively. LO had the highest influence on globule size, whereas the stability index was most influenced by Lauroglycol-FCC. The optimized formula contained 14% LO, 40.5% Labrasol:Labrafil 1944 in the ratio of 6:4, 45.5% Lauroglycol-FCC, and 100 mg PV. The rheological characterization study of the optimized gel (O3) revealed its combined pseudoplastic and thixotropic behavior. The permeation studies in sheep buccal mucosa confirmed that the O3 formulation can significantly improve PV permeation, while the pharmacokinetic data indicate that O3 can enhance the bioavailability of PV and provide a sustained release. Altogether, these findings validate the usefulness of the O3 formulation, containing the optimized PV-LO-SNEDDS formulation in chitosan hydrogel, as a promising therapeutic approach against herpes labialis.
